# Biological age acceleration and mortality risk among U.S. adults with cardio-kidney-metabolic syndrome, stages 0–3: An NHANES cohort study

**DOI:** 10.1097/MD.0000000000050450

**Published:** 2026-08-28

**Authors:** Xiaoqiang Chen, Yisen Huang, Yingxuan Huang, Yingyi Li, Xinqi Chen, Yubin Wang, Xiaoqiang Liu

**Affiliations:** a Department of Otolaryngology, Fujian Medical University Union Hospital, Fuzhou, Fujian, China; b Department of Gastroenterology, First Hospital of Quanzhou Affiliated to Fujian Medical University, Quanzhou, Fujian, China.

**Keywords:** Biological Age Acceleration, CKM syndrome, Klemera-Doubal Method (KDM-BA), NHANES, Phenotypic Age (PA)

## Abstract

This study aims to evaluate the associations of Klemera-Doubal Method Biological Age Acceleration (KDM-BAA) and Phenotypic Age Acceleration (PAA) with all-cause and cause-specific mortality in U.S. adults with Cardio-Kidney-Metabolic (CKM) syndrome stages 0–3 and to establish risk prediction models. Data were obtained from 16,837 participants in NHANES 1999–2010 and 2015–2018, linked to mortality through 2019. Biological age was estimated using KDM-BA and Phenotypic Age algorithms, with residuals defining Biological Age Acceleration (BAA). Cox proportional hazards models were applied, and LASSO-Cox nomograms were developed for mortality prediction. Over a median follow-up of 11.33 years, 10.8% of the weighted population (9.75 million adults) died. After full adjustment for confounders, KDM-BAA positivity increased the risk of all-cause, CVD, and non-CVD mortality by 41%, 74%, and 31%, respectively; PAA positivity showed even larger increases – 103%, 79%, and 112%. Both metrics

consistently exhibited a positive association in multiple subgroups and sensitivity analyses. The LASSO-Cox-based nomogram showed favorable discrimination (training AUCs 0.776 and 0.775 at 10 and 20 years; validation AUCs 0.765 and 0.757, respectively) and good calibration, suggesting potential clinical applicability. BAA independently predicts long-term mortality among individuals with CKM stages 0–3. PAA provides superior prognostic value, supporting the importance of monitoring biological aging to guide early intervention and improve long-term outcomes.

## 1. Introduction

With population aging and the shifting spectrum of chronic diseases, the integrated prevention and control of cardiovascular, kidney, and metabolic abnormalities face severe challenges.^[1]^ In 2023, the American Heart Association (AHA) proposed an updated Cardio-Kidney-Metabolic (CKM) syndrome staging framework to systematically evaluate the progression from obesity or metabolic dysfunction alone to clinical cardiovascular events and kidney failure.^[2]^ Although stages 0–3 are often regarded as low risk, evidence shows that even early metabolic abnormalities or mild renal impairment may be accompanied by subclinical inflammation and immune dysregulation, conferring elevated risks of future cardiovascular and all-cause mortality.^[3,4]^ Renal impairment is not merely a component of CKM staging but an independent determinant of survival, predicting long-term mortality even after an acute cardiovascular event.^[5]^

Chronological age is a crude proxy for physiological reserve: among patients of comparable age with established cardiovascular disease, outcomes diverge with cardiac, renal, and laboratory profile,^[6]^ and mortality prediction scores perform inconsistently in older populations.^[7]^ Biological age has emerged as a key marker linking aging processes to cardiovascular risk. Beyond epigenetic clocks such as GrimAge, the Klemera-Doubal method (KDM-BA) and Phenotypic Age (PA) estimate biological age from routine physiological and biochemical indices, including blood pressure, glucose, creatinine, C-reactive protein, and red cell distribution width.^[8,9]^ A recent study in CKM populations demonstrated that each 5-unit increase in GrimAge was associated with substantially higher all-cause and cardiovascular mortality risk, underscoring the prognostic value of epigenetic clocks.^[10]^ However, their cost and technical requirements limit their feasibility in routine care and in large-scale epidemiological research, whereas KDM-BA and PA can be derived from measurements already obtained in standard clinical practice.

It is important to distinguish 2 lines of inquiry. A substantial body of work has established that a higher CKM stage predicts greater mortality, treating CKM staging itself as the exposure. The present study addresses a different question: within a population already confined to CKM stages 0–3, does the extent to which an individual’s biological age exceeds their chronological age carry prognostic information? Here CKM staging serves as an eligibility criterion rather than an exposure, and biological age acceleration (BAA) is the exposure of interest. This distinction matters because stages 0–3 encompass individuals with obesity, metabolic abnormality, or early renal impairment but no clinical cardiovascular event, a group conventionally regarded as low risk and therefore seldom prioritized for intensive intervention. Previous evidence that BAA predicts mortality has come largely from older adults or from patients with established chronic disease such as diabetes, heart failure, or end-stage renal disease; whether it retains prognostic value in nationally representative populations at the early end of the cardiorenal-metabolic continuum remains largely untested.

Accordingly, using eight cycles of the National Health and Nutrition Examination Survey (NHANES), we assessed BAA by KDM-BA and PA and examined its associations with all-cause, cardiovascular, and non-cardiovascular mortality among adults in CKM stages 0–3. We further developed and validated a mortality risk prediction model, aiming to improve early risk stratification in this population and to support the clinical application of biological age monitoring.

## 2. Methods

### 2.1. Study design

**Figure 1.**
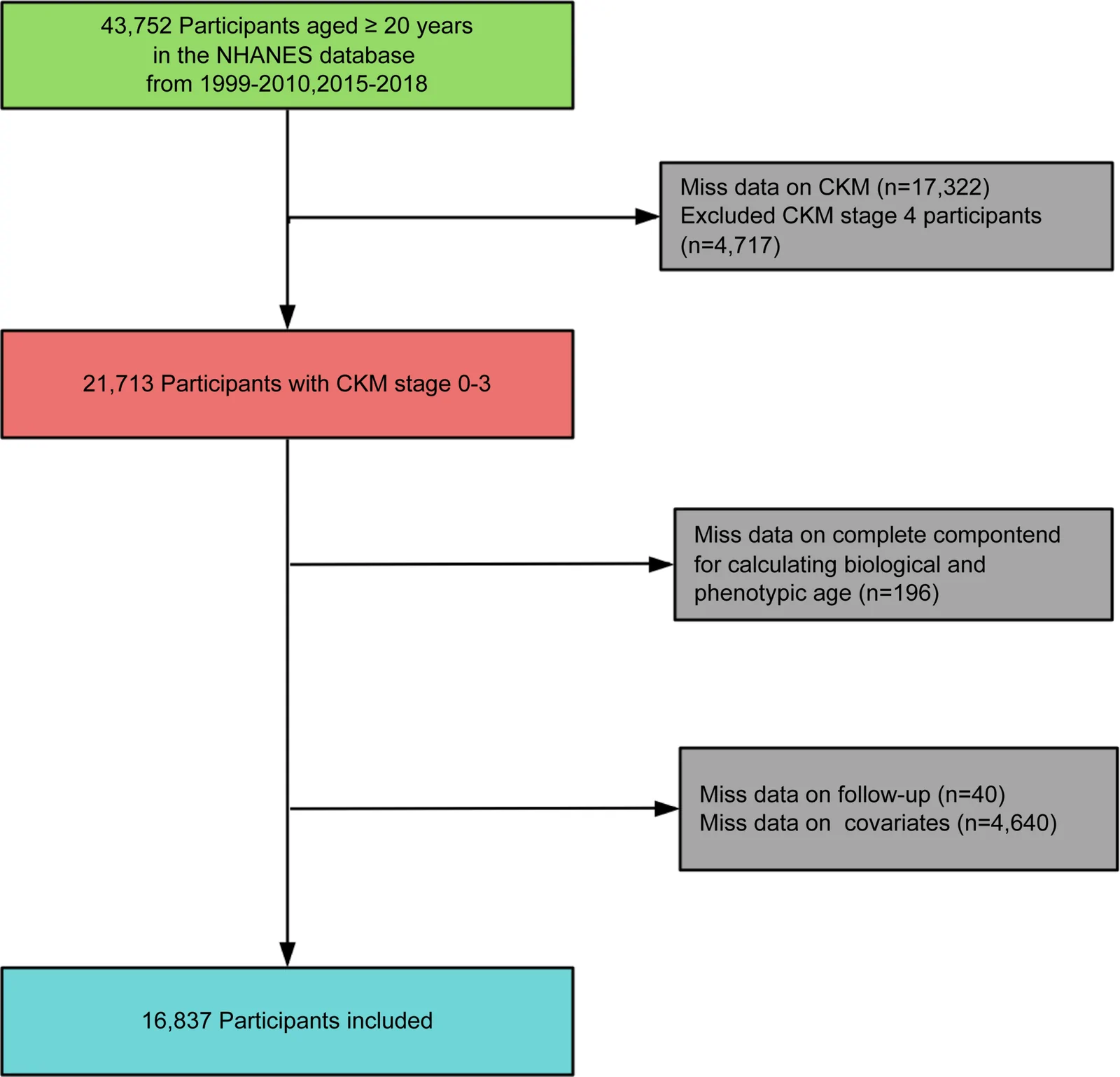
Flowchart of the study participant selection process. CKM = cardiovascular-kidney-metabolic.

This study utilized cross-sectional and prospective follow-up data from the NHANES spanning eight cycles (1999–2010, 2015–2018), with mortality outcomes traced via the National Death Index (NDI) through December 31, 2019. The 2011–2014 cycles were excluded because they lacked CRP data necessary to calculate Phenotypic Age. NHANES employs a stratified, multi-stage, probability sampling design that covers the noninstitutionalized U.S. population. All participants provided written informed consent, and the program was approved by the National Center for Health Statistics (NCHS) Research Ethics Review Board. As this study analyzed only publicly available, de-identified data, additional ethical approval was deemed unnecessary. The participant selection flowchart is shown in Figure 1. Starting from 43,752 NHANES respondents aged ≥ 20 years across eight cycles, we first excluded 17,322 individuals lacking CKM information and 4717 individuals already in CKM stage 4, leaving 21,713 participants who met CKM stages 0–3. Then, we excluded 196 participants lacking complete biochemical data required to calculate biological age, 40 participants without mortality follow-up information, and 4640 participants with missing key covariates. Ultimately, 16,837 participants were included in the final analytic cohort.

### 2.2. Definition of CKM Stages 0–3

Referring to the 2023 AHA framework for CKM syndrome staging, participants in NHANES were categorized into stages 0–4: stage 0 indicates no overweight/obesity, metabolic risk factors, chronic kidney disease (CKD), or subclinical/clinical cardiovascular disease (CVD); stage 1 indicates overweight, abdominal obesity, or adipose tissue dysfunction without any other metabolic risk factors or CKD; stage 2 indicates ≥ 1 metabolic risk factor such as hypertension, dyslipidemia, or impaired glucose regulation, and/or early CKD; stage 3 indicates subclinical CVD (or extremely high predicted risk) and/or moderate-to-severe CKD, suggesting a significantly elevated absolute cardiovascular risk; stage 4 indicates clinically confirmed CVD (including coronary artery disease, heart failure, stroke, peripheral arterial disease, or atrial fibrillation) with or without kidney failure, further divided into 4a (no kidney failure) and 4b (with kidney failure). If a participant met criteria for multiple stages, they were classified into the highest stage.^[2]^ Detailed definitions are provided in Table S1, Supplemental Digital Content 1. This study focused on CKM stages 0–3, thus excluding all participants at stage 4.

### 2.3. Assessment of biological age acceleration

KDM-BA is derived via weighted linear regression using ten common laboratory and physiological indices identified by Levine and colleagues. These indices are systolic blood pressure (SBP), creatinine, glycated hemoglobin (HbA1c), CRP, albumin, alkaline phosphatase (ALP), white blood cell count (WBC), mean corpuscular volume (MCV), lymphocyte percentage, and pulse rate.^[11]^ PA is computed with the original formula from the same group and incorporates nine variables, namely chronological age, albumin, creatinine, glucose, lymphocyte percentage, mean corpuscular volume, RDW, CRP, and alkaline phosphatase.^[12]^ Further details on both calculations are provided in the Supplementary Methods, Supplemental Digital Content 2. For both methods, we took the residual of (biological age - chronological age). A residual greater than zero was classified as accelerated, defined as Klemera-Doubal Method Biological Age Acceleration (KDM-BAA) or Phenotypic Age Acceleration (PAA), whereas a residual of zero or less was classified as non-accelerated. In dose-response analyses, continuous residuals were entered into the model and scaled per 1 standard deviation increase.

### 2.4. Definition of outcomes

Mortality data were obtained from the NDI. Outcome events comprised 3 categories: all-cause mortality; cardiovascular mortality, defined by ICD-10 codes I00-I99; non-cardiovascular mortality, referring to all deaths excluding I00-I99. Follow-up time began on the date of baseline interview and ended at death, loss to follow-up, or December 31, 2019, whichever came first.

### 2.5. Definition of covariates

Covariate selection and definitions were based on previous studies and clinical judgment.^[13,14]^ Demographic and socioeconomic variables included chronological age (continuous), sex, race (Non-Hispanic White, Non-Hispanic Black, Mexican American or Other), marital status (married or living with partner versus never married or other), poverty-income ratio (PIR) grouped as < 1.3, 1.3–3.5 or > 3.5, and education level (< high school, high school or equivalent, > high school). Lifestyle factors comprised smoking status classified as never (< 100 cigarettes in a lifetime), former (≥100 cigarettes but not currently smoking) or current^[15]^; alcohol intake categorized as never (<12 drinks in a lifetime), former (≥12 drinks in any year but none in the past year or no drinks in the past year with ≥ 12 drinks lifetime) or current (≥12 drinks in any year and alcohol consumed in the past year)^[16]^; weekly physical activity (PA) time (continuous) from walking, cycling, occupational and recreational activities, further divided into inactive (0 MET-min/wk), insufficiently active (1–599 MET-min/wk) and sufficiently active (≥600 MET-min/wk)^[17]^; and diet quality measured by the Healthy Eating Index-2015 (HEI-2015) score (continuous).^[18]^ Clinical characteristics included body mass index (BMI, calculated as weight in kilograms divided by height in meters squared) and current use (yes or no) of antihypertensive, antidiabetic or lipid-lowering medications as indicators of treated hypertension, diabetes and hyperlipidemia, respectively.

### 2.6. Statistical analysis

Given NHANES’s complex sampling design, all analyses applied a survey framework specifying sampling weights, strata, and primary sampling units to yield nationally representative estimates. For the fasting subsample in 1999–2010 and 2015–2018, we used the NHANES-provided “WTSAF4YR” or “WTSAF2YR” weights, then adjusted for each cycle’s duration (weights for 1999–2000 and 2001–2002 were set as 2/8 × WTSAF4YR, and 1/8 × WTSAF2YR for the other cycles), to ensure integrated results representing the target U.S. population appropriately. Baseline characteristics are reported as weighted percentages for categorical variables and weighted means ± standard errors for continuous variables. Group differences were tested by weighted chi-square tests for categorical variables and weighted independent-samples *t*-tests for continuous variables.

The associations between outcomes and follow-up duration were quantified using survey-weighted Cox proportional hazards models. Covariate inclusion was guided by clinical expertise, univariate screening (*P* < .05), and a change-in-estimate criterion >10%. We also assessed multicollinearity with variance inflation factors (VIFs) and found none, as every VIF was below 2.0, well beneath the conventional threshold of 5. We incrementally adjusted for covariates in 3 steps: Model 1: adjusted for age and sex only. Model 2: further adjusted for race, marital status, PIR, education level, BMI, HEI-2015, physical activity, smoking status, and alcohol consumption. Model 3: additionally adjusted for medication use for diabetes, hypertension, and hyperlipidemia. Proportional hazards assumptions were assessed by the Schoenfeld residual test. If assumptions held, weighted Kaplan–Meier curves were plotted, and the log-rank test was used to compare survival differences between exposure groups. To explore dose-response relationships, restricted cubic splines (RCS) with 3 knots (5th, 50th, 95th percentiles) were applied to visualize potential nonlinear associations between biological age residuals and mortality risk, and to test for overall trend. To confirm the generalizability of the main results, stratified analyses were performed by prespecified subgroups, including age, sex, smoking, alcohol intake, physical activity, and medication status. Interaction terms were tested. Sensitivity analyses excluded participants who died within the first 2 years of follow-up and further adjusted for CKM stage to gauge the robustness of the findings.

For prediction model development, the sample was randomly split 7:3 into a training set and a validation set. LASSO-Cox regression (10-fold cross-validation, using minimum λ) was employed in the training set for variable selection and nomogram construction. Time-dependent C-index/AUC and 1000 bootstrap calibration curves were used to evaluate the model’s discrimination and calibration. We used decision curve analysis to assess net clinical benefits across different threshold probabilities and determine the model’s potential application value. All analyses were carried out using R (http://www.R-project.org, R Foundation) and Free Statistics software (version 2.1.1, Beijing, China). A two-sided *P* < .05 was considered statistically significant.

## 3. Results

### 3.1. Study population and baseline characteristics

As shown in Table 1, this study ultimately included a nationally representative sample of 90,153,719 U.S. adults (weighted) with CKM stages 0–3, with a mean age of 47.6 ± 16.0 years and 50.9% female. Compared to those without KDM-BAA, individuals with KDM-BAA were older (50.88 vs 44.55 years), had a higher proportion of males (56.16% vs 41.67%), higher BMI (30.96 vs 27.43 kg/m^2^), and were more likely to be on medications for hyperglycemia, dyslipidemia, and hypertension (all *P* < .001). The PAA-positive group showed a similar pattern, suggesting that those with BAA have more severe metabolic/cardiorenal risk exposures overall.

**Table 1:** Baseline characteristics of individuals.

Characteristics	Overall	KDM–BAA		*P* value	PAA		*P* value
		No	Yes		No	Yes	
**Weighted Population, n**	90153719	44041947	46111772		62198890	27954829	
**Age, mean (SD**)	47.64 (16.04)	50.88 (15.61)	44.55 (15.84)	<.001	46.92 (15.95)	49.25 (16.13)	<.001
**Sex (%**)				<.001			<.001
Male	44248896 (49.08)	18351304 (41.67)	25897592 (56.16)		29461408 (47.37)	14787488 (52.90)	
Female	45904823 (50.92)	25690643 (58.33)	20214180 (43.84)		32737482 (52.63)	13167341 (47.10)	
**Race, n (%**)				<.001			<.001
Non–Hispanic White	62522063 (69.35)	32982738 (74.89)	29539325 (64.06)		44863188 (72.13)	17658875 (63.17)	
Non–Hispanic Black	9728601 (10.79)	3513025 (7.98)	6215575 (13.48)		5623780 (9.04)	4104821 (14.68)	
Mexican American	7525359 (8.35)	3150945 (7.15)	4374414 (9.49)		4885010 (7.85)	2640349 (9.45)	
Other Race	10377696 (11.51)	4395238 (9.98)	5982458 (12.97)		6826913 (10.98)	3550783 (12.70)	
**Marital status, n (%**)				<.001			<.001
Married/ Living with partner	59605631 (66.12)	30008387 (68.14)	29597244 (64.19)		42344623 (68.08)	17261008 (61.75)	
Never married/Other	30548088 (33.88)	14033560 (31.86)	16514528 (35.81)		19854268 (31.92)	10693821 (38.25)	
**PIR, n (%**)				<.001			<.001
1–1.3	17108231 (18.98)	7439593 (16.89)	9668638 (20.97)		10494166 (16.87)	6614065 (23.66)	
1.31–3.50	32678704 (36.25)	15508433 (35.21)	17170271 (37.24)		22335942 (35.91)	10342762 (37.00)	
>3.50	40366784 (44.78)	21093921 (47.90)	19272864 (41.80)		29368782 (47.22)	10998002 (39.34)	
**Education level, n (%**)				.034			.005
Less than high school	15252514 (16.92)	7266398 (16.50)	7986116 (17.32)		10296510 (16.55)	4956004 (17.73)	
High school or equivalent	22411234 (24.86)	10478929 (23.79)	11932305 (25.88)		14941154 (24.02)	7470080 (26.72)	
Above high school	52489971 (58.22)	26296620 (59.71)	26193351 (56.80)		36961226 (59.42)	15528745 (55.55)	
**BMI, mean (SD), kg/m** ^**2**^	29.23 (6.84)	27.43 (5.82)	30.96 (7.28)	<.001	27.93 (5.86)	32.14 (7.88)	<.001
**HEI–2015 score, mean (SD**)	49.95 (13.29)	51.33 (13.40)	48.63 (13.04)	<.001	50.80 (13.31)	48.05 (13.04)	<.001
**Smoking status, n (%**)				.016			<.001
Never	47765058 (52.98)	22697679 (51.54)	25067379 (54.36)		33997349 (54.66)	13767709 (49.25)	
Former	23258653 (25.80)	11815511 (26.83)	11443142 (24.82)		15926400 (25.61)	7332253 (26.23)	
Current	19130008 (21.22)	9528757 (21.64)	9601251 (20.82)		12275142 (19.74)	6854866 (24.52)	
**Alcohol intake, n (%**)				<.001			.132
Never	9968442 (11.06)	5031328 (11.42)	4937114 (10.71)		7101620 (11.42)	2866822 (10.26)	
Former	13304324 (14.76)	7145019 (16.22)	6159305 (13.36)		9299014 (14.95)	4005310 (14.33)	
Current	66880953 (74.19)	31865600 (72.35)	35015353 (75.94)		45798256 (73.63)	21082697 (75.42)	
**Physical activity, n (%**)				<.001			<.001
Inactive	20991549 (23.28)	10357479 (23.52)	10634070 (23.06)		13902697 (22.35)	7088852 (25.36)	
Insufficiently active	46365934 (51.43)	24218284 (54.99)	22147650 (48.03)		34434087 (55.36)	11931847 (42.68)	
Sufficiently active	22796236 (25.29)	9466184 (21.49)	13330052 (28.91)		13862106 (22.29)	8934130 (31.96)	
**Antidiabetic drugs, n (%**)				<.001			<.001
No	81620698 (90.54)	41277969 (93.72)	40342729 (87.49)		59218375 (95.21)	22402323 (80.14)	
Yes	8533021 (9.46)	2763977 (6.28)	5769044 (12.51)		2980515 (4.79)	5552506 (19.86)	
**Antihyperlipidemic drugs, n (%**)				<.001			<.001
No	76714018 (85.09)	36551548 (82.99)	40162470 (87.10)		54298488 (87.30)	22415530 (80.18)	
Yes	13439701 (14.91)	7490399 (17.01)	5949302 (12.90)		7900402 (12.70)	5539299 (19.82)	
**Antihypertensive drugs, n (%**)				.013			<.001
No	66937672 (74.25)	33254594 (75.51)	33683078 (73.05)		48837413 (78.52)	18100259 (64.75)	
Yes	23216047 (25.75)	10787352 (24.49)	12428695 (26.95)		13361477 (21.48)	9854570 (35.25)	
**CKM stage, n (%**)				<.001			<.001
0	9009421 (9.99)	6665145 (15.13)	2344276 (5.08)		8133860 (13.08)	875561 (3.13)	
1	17113368 (18.98)	10400492 (23.61)	6712876 (14.56)		13135538 (21.12)	3977830 (14.23)	
2	56111325 (62.24)	23230140 (52.75)	32881185 (71.31)		36976032 (59.45)	19135293 (68.45)	
3	7919605 (8.78)	3746169 (8.51)	4173436 (9.05)		3953461 (6.36)	3966144 (14.19)	

Data were presented as weighted percentages or means (SD).

BMI = body mass index, CKM = cardiovascular-kidney-metabolic, HEI-2015 = Healthy Eating Index-2015, KDM-BAA = Klemera-Doubal Method Biological Age Acceleration, PAA = phenotypic age acceleration, PIR = poverty-income ratio.

### 3.2. Association between biological age acceleration and all-cause/cause-specific mortality

During the median follow-up of 11.33 years (IQR: 4.25–15.58), weighted analysis indicated a total of about 9,754,249 deaths (10.8%), including 2,635,057 (2.9%) from CVD and 7,119,191 (7.9%) from non-CVD causes. As shown in Table 2, in unadjusted models, KDM-BAA was not significantly associated with all-cause mortality (HR = 1.00, 95% CI 0.91–1.10), whereas PAA positivity elevated mortality risk by about twofold (HR = 2.84, 95% CI 2.55–3.17). After sequentially controlling for demographic factors, lifestyle variables, diet quality, and medication use, both measures emerged as independent predictors of all-cause and cause-specific mortality. Specifically, KDM-BAA positivity increased all-cause mortality risk by 41% (HR = 1.41, 95% CI 1.29–1.54), CVD mortality by 74% (HR = 1.74, 95% CI 1.46–2.09), and non-CVD mortality by 31% (HR = 1.31, 95% CI 1.19–1.44). PAA positivity showed more pronounced increases: 103% for all-cause mortality (HR = 2.03, 95% CI 1.82–2.26), 79% for CVD mortality (HR = 1.79, 95% CI 1.46–2.18), and 112% for non-CVD mortality (HR = 2.12, 95% CI 1.87–2.41). When treated as continuous variables, each 1-SD increase in KDM-BAA residual was associated with a 5% rise in all-cause mortality (HR = 1.05, 95% CI 1.04–1.06), and each 1-SD increase in PAA residual led to a 4% rise (HR = 1.04, 95% CI 1.03–1.04).

**Table 2:** Weighted adjusted hazard ratios of biological aging with risk of all–cause and cause–specific mortality among adults with CKM stages 0–3.

Characteristics	Crude model		Model 1		Model 2		Model 3	
	HR (95% CI)	*P* value	HR (95% CI)	*P* value	HR (95% CI)	*P* value	HR (95% CI)	*P* value
**All–cause mortality**								
KDM–BAA residual	1.01 (1.00, 1.03)	.097	1.05 (1.04, 1.06)	<.001	1.05 (1.04, 1.06)	<.001	1.05 (1.04, 1.06)	<.001
KDM–BAA								
No	Reference		Reference		Reference		Reference	
Yes	1.00 (0.91, 1.10)	.956	1.53 (1.41, 1.66)	<.001	1.48 (1.35, 1.62)	<.001	1.41 (1.29, 1.54)	<.001
PAA residual	1.04 (1.04, 1.05)	<.001	1.04 (1.04, 1.05)	<.001	1.04 (1.03, 1.04)	<.001	1.04 (1.03, 1.04)	<.001
PAA								
No	Reference		Reference		Reference		Reference	
Yes	2.84 (2.55, 3.17)	<.001	2.40 (2.16, 2.66)	<.001	2.11 (1.89, 2.35)	<.001	2.03 (1.82, 2.26)	<.001
**CVD mortality**								
KDM–BAA residual	1.04 (1.02, 1.07)	.001	1.08 (1.06, 1.10)	<.001	1.07 (1.05, 1.09)	<.001	1.06 (1.04, 1.08)	<.001
KDM–BAA								
No	Reference		Reference		Reference		Reference	
Yes	1.24 (1.04, 1.48)	.016	2.04 (1.72, 2.41)	<.001	1.89 (1.58, 2.28)	<.001	1.74 (1.46, 2.09)	<.001
PAA residual	1.04 (1.04, 1.05)	<.001	1.04 (1.04, 1.05)	<.001	1.04 (1.03, 1.04)	<.001	1.03 (1.02, 1.04)	<.001
PAA								
No	Reference		Reference		Reference		Reference	
Yes	2.92 (2.44, 3.48)	<.001	2.40 (2.00, 2.88)	<.001	2.00 (1.65, 2.43)	<.001	1.79 (1.46, 2.18)	<.001
**Non–CVD mortality**								
KDM–BAA residual	1.00 (0.98, 1.02)	.815	1.04 (1.03, 1.06)	<.001	1.04 (1.03, 1.06)	<.001	1.04 (1.03, 1.05)	<.001
KDM–BAA								
No	Reference		Reference		Reference		Reference	
Yes	0.92 (0.83, 1.02)	.129	1.38 (1.25, 1.51)	<.001	1.36 (1.23, 1.49)	<.001	1.31 (1.19, 1.44)	<.001
PAA residual	1.04 (1.04, 1.05)	<.001	1.04 (1.04, 1.05)	<.001	1.04 (1.03, 1.04)	<.001	1.04 (1.03, 1.04)	<.001
PAA								
No	Reference		Reference		Reference		Reference	
Yes	2.81 (2.48, 3.18)	<.001	2.39 (2.12, 2.70)	<.001	2.15 (1.90, 2.43)	<.001	2.12 (1.87, 2.41)	<.001

Crude model: no other covariates were adjusted.

Model 1: Adjusted for age and sex.

Model 2: Further adjusted for race, marital status, PIR, education, BMI, smoking status, alcohol intake, physical activity, and HEI-2015.

Model 3: Further adjusted for antidiabetic drugs, antihyperlipidemic drugs, and antihypertensive drugs.

BMI = body mass index, CKM = cardiovascular-kidney-metabolic, HEI-2015 = Healthy Eating Index- 2015, KDM-BAA = Klemera-Doubal Method Biological Age Acceleration, PAA = phenotypic age acceleration, PIR = poverty-income ratio.

**Figure 2.**
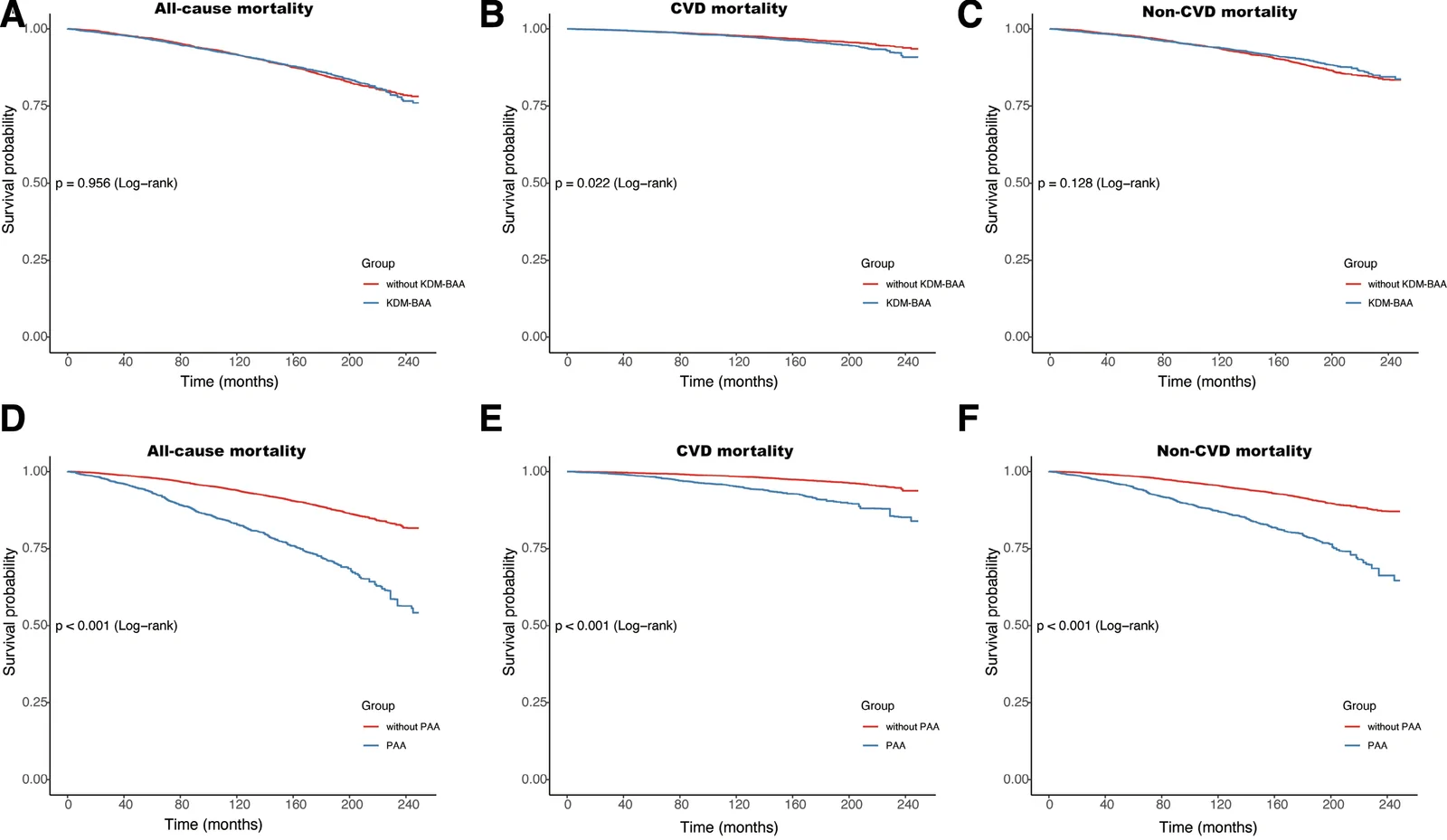
Survey-weighted Kaplan–Meier survival curves for all-cause, CVD, and Non-CVD mortality according to KDM biological age acceleration (Panels A-C) and phenotypic age acceleration (Panels D-F) in adults with CKM stages 0–3. KDM-BAA = Klemera-Doubal Method Biological Age Acceleration, PAA = phenotypic age acceleration, CKM = cardiovascular-kidney-metabolic.

**Figure 3.**
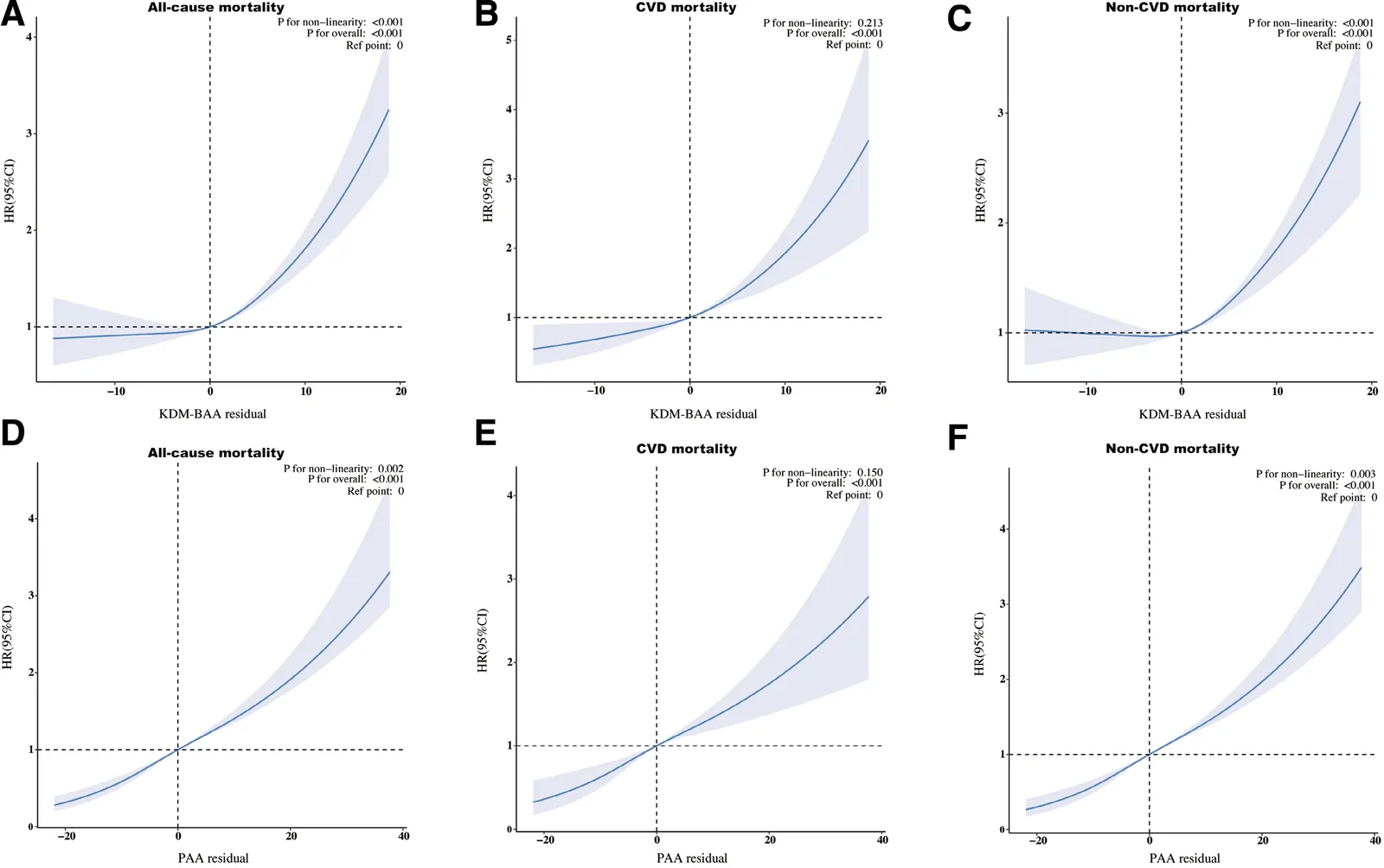
Survey-weighted dose-response associations for all-cause, CVD, and non-CVD mortality according to KDM biological age acceleration residual (Panels A–C) and phenotypic age acceleration residual (Panels D-F) in adults with CKM stages 0–3. This spline model was adjusted for age, sex, race, marital status, PIR, education, BMI, smoking status, alcohol intake, physical activity, HEI-2015, antidiabetic drugs, antihyperlipidemic drugs, and antihypertensive drugs. PIR = poverty-income ratio, BMI = body mass index, HEI-2015 = Healthy Eating Index −2015, KDM = Klemera-Doubal Method; CKM, cardiovascular-kidney-metabolic.

As shown in the Kaplan–Meier weighted curves (Fig. 2), the survival trajectories for the KDM-BAA positive versus negative groups were virtually identical for all-cause and non-CVD mortality (log-rank test, *P* = .956 and *P* = .128, respectively), with only a slight divergence observed for CVD mortality (*P* = .022). By contrast, the PAA-positive group exhibited an immediate and progressively widening survival disadvantage across all three mortality endpoints (all *P* < .001), indicating that PAA is superior to KDM-BAA in distinguishing and predicting multiple forms of death. RCS demonstrated a roughly linear dose-response relationship between both KDM-BAA and PAA residuals and the risk of all-cause, CVD, and non-CVD mortality, with no obvious threshold effects (Fig. 3).

### 3.3. Subgroup and sensitivity analyses

**Figure 4.**
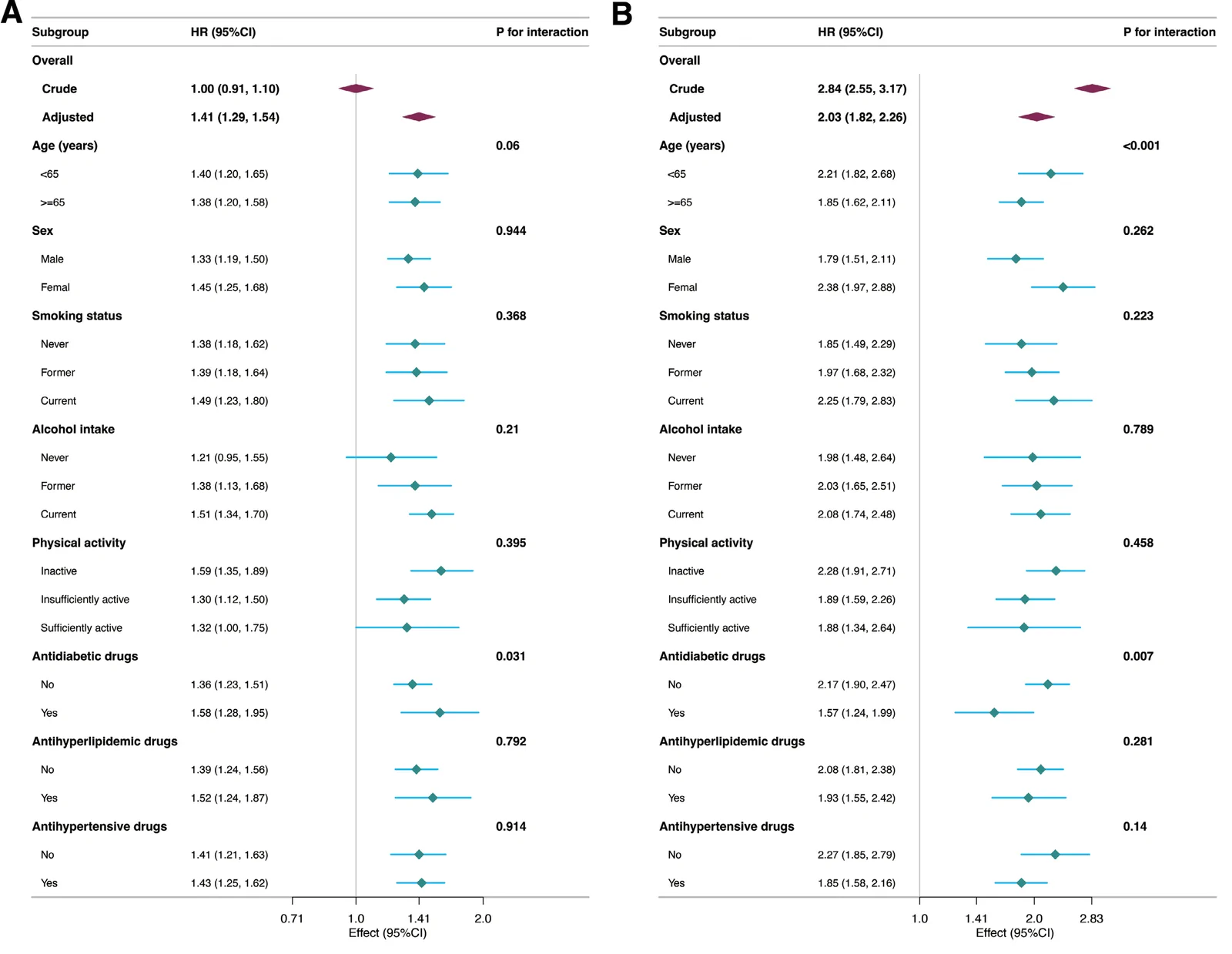
Subgroup analyses of the association of the KDM biological age acceleration (A) and phenotypic age acceleration (B) with all-cause mortality among adults with CKM stages 0–3. This model was adjusted for age, sex, race, marital status, PIR, education, BMI, smoking status, alcohol intake, physical activity, HEI-2015, antidiabetic drugs, antihyperlipidemic drugs, and antihypertensive drugs. PIR = poverty-income ratio, BMI = body mass index, HEI-2015 = Healthy Eating Index-2015, KDM = Klemera-Doubal Method, CKM = cardiovascular-kidney-metabolic.

In prespecified subgroups categorized by age, sex, smoking, alcohol intake, physical activity, and medication status (Fig. 4), as well as in continuous-residual form (Fig. S1, Supplemental Digital Content 3), both KDM-BAA and PAA displayed the same direction of effect on all-cause mortality, with no significant interactions observed. This implies consistent and robust associations across different participant characteristics.

When participants who died within 2 years of follow-up were excluded (Table S2, Supplemental Digital Content 4), the effect estimates slightly weakened but remained significant (HR = 1.35 for KDM-BAA positivity, HR = 1.95 for PAA positivity). Further adjustment for CKM stage (Table S3, Supplemental Digital Content 5) yielded HR = 1.34 (95% CI 1.22–1.46) for KDM-BAA positivity and HR = 1.97 (95% CI 1.77–2.20) for PAA positivity, confirming the independence of BAA beyond CKM severity.

### 3.4. Construction and validation of prediction models

**Figure 5.**
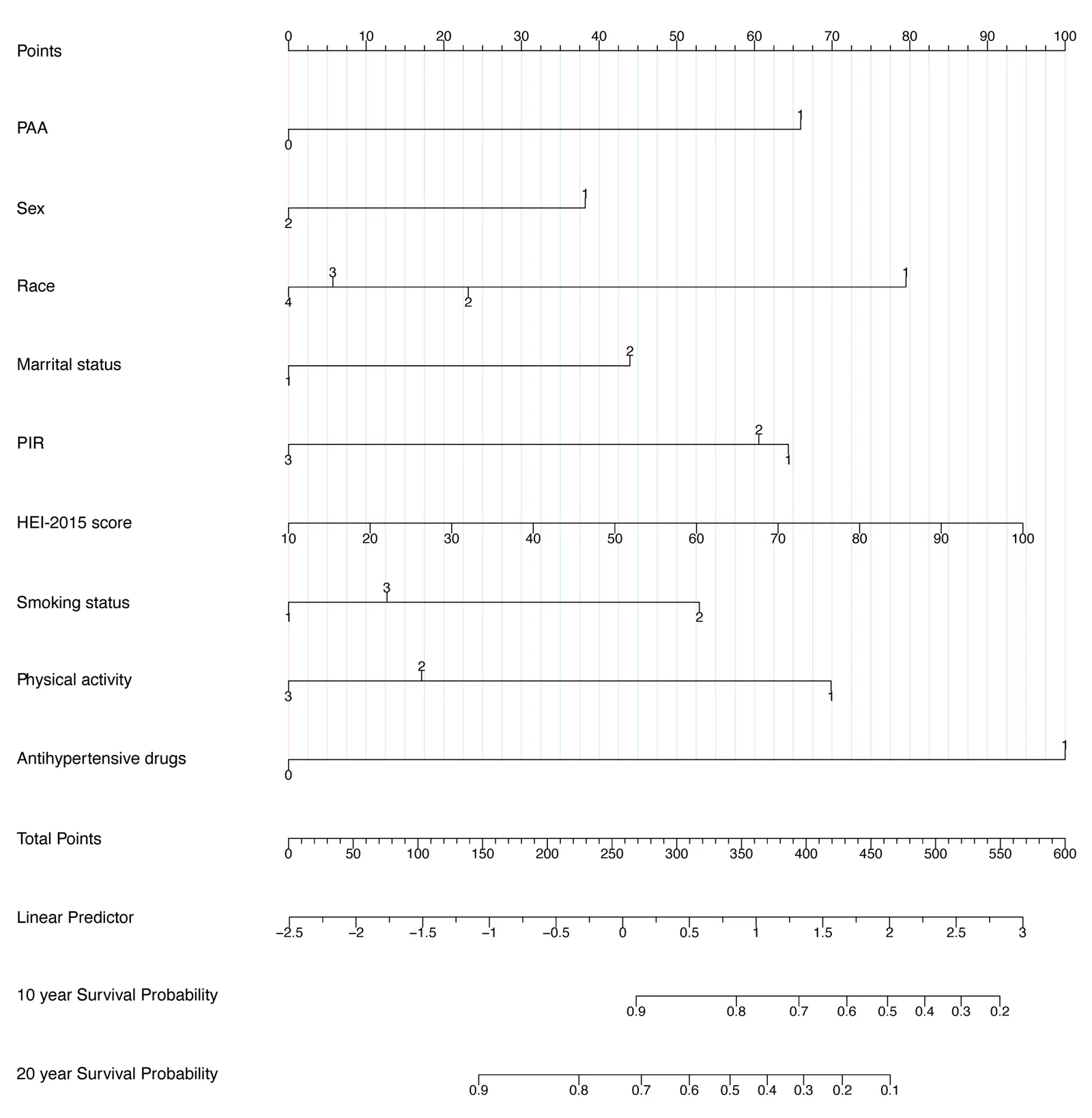
Nomogram for predicting the risk of all-cause mortality. The first line represents the scoring scale. Corresponding scores for each predictor factor are shown in lines 2–10. The score for each predictor is determined by referencing the first line. The total score for the risk evaluation is the sum of each predictor score. To determine the likelihood of all-cause mortality, the score point is located on the total point line (line 11). Then, the user descends vertically to the risk of all-cause mortality (lines 13 and 14). Numbers in the figure represent the following values: sex:1 = male, 2 = female. Race: 1 = non-Hispanic white, 2 = non-Hispanic black, 3 = Mexican American, 4 = other race. Marital status: 1 = married/ living with partner, 2 = never married/other. Smoking status: 1 = never, 2 = former, 3 = current. Physical activity:1** = **inactive, 2 = insufficiently active, 3 = sufficiently active. PIR: 1 = 1-1.3, 2 = 1.31-3.50, 3= >3.50. Antihypertensive drugs: 0 = no, 1 = yes. PIR = poverty-income ratio, HEI-2015 = Healthy Eating Index −2015, PAA = phenotypic age acceleration.

In the training set, LASSO-Cox regression (10-fold cross-validation with minimum λ) identified nine nonzero coefficient features out of 14 candidate variables (Fig. S2, Supplemental Digital Content 6). A nomogram based on these features (Fig. 5) provided straightforward estimates of individual 10-year and 20-year all-cause mortality probabilities. Time-dependent ROC curves indicated that in the training set, the model achieved AUCs of 0.776 and 0.775 at 10 and 20 years, respectively, and 0.765 and 0.757 in the validation set, all surpassing the clinically acceptable threshold of 0.75 (Fig. S3, Supplemental Digital Content 7). Decision curve analysis demonstrated a clear net benefit for threshold probabilities of 6–57% (10-year) and 14–75% (20-year) in the training set, and 6–51% (10-year) and 12–61% (20-year) in the validation set (Fig. S4, Supplemental Digital Content 8). Additionally, calibration curves in both datasets closely approximated the ideal 45° line, reflecting a high degree of concordance between predicted and observed event rates (Fig. S5, Supplemental Digital Content 9).

## 4. Discussion

Drawing on a nationally representative NHANES sample, this study systematically examined the association between biological age acceleration (KDM-BAA and PAA) and risks of all-cause and cause-specific mortality in adults with CKM syndrome stages 0–3. The findings indicate that both the KDM-BA- and PA-based residual definitions of BAA correlate significantly with elevated mortality. However, PAA showed stronger predictive capacity for all-cause, CVD, and non-CVD mortality compared to KDM-BAA. Subgroup, sensitivity, and dose-response analyses revealed no obvious subgroup differences or threshold phenomena, and the associations remained robust after extensive adjustment. Given the relative scarcity of research on mortality prediction tools specifically in early-stage CKM cohorts, our results not only enhance current approaches to disease staging but also offer novel insights into more precise risk identification and earlier intervention in clinical and public health contexts.

This study focused on CKM syndrome stages 0–3, employing two commonly used biochemical-based biological age algorithms (KDM-BA and PA). Both effectively predicted all-cause and cause-specific mortality. Notably, in the unadjusted model, KDM-BAA was not significantly associated with all-cause mortality, but once potential confounders such as demographics, lifestyle, and medication use were fully controlled, its predictive ability emerged. By contrast, from the crude model onward, PAA demonstrated a persistently significant influence on all-cause and cardiovascular/non-cardiovascular mortality, with hazard ratios visibly exceeding those for KDM-BAA. Kaplan–Meier survival analysis likewise corroborated this distinction, revealing that while the KDM-BAA positive and negative groups showed little divergence in all-cause or non-cardiovascular mortality, the PAA-positive group separated from its negative counterpart early in follow-up with a steadily growing survival disadvantage. This distinction between PAA and KDM-BAA aligns with an increasing number of recent publications.^[19,20]^

Earlier studies primarily relied on KDM-BA to evaluate physiological biological aging from blood pressure, glucose, lipids, creatinine, and other routine clinical markers. However, given the burgeoning recognition of chronic inflammation’s central role in cardiovascular, renal, and metabolic diseases, more researchers have begun integrating CRP, RDW, and other immunological parameters into aging clock models to better capture the additional risk imposed by chronic inflammatory and immunosenescence processes.^[21]^ PA, proposed by Levine et al., exemplifies this approach and has consistently shown superior prediction of mortality and cardiovascular outcomes in several prospective cohorts.^[22,23]^ Our study reaffirms these findings: in fully adjusted models, PAA demonstrated more potent and more stable mortality discrimination than KDM-BAA.

Moreover, unlike many earlier studies that predominantly focused on older adults or individuals already diagnosed with chronic conditions such as diabetes, heart failure, or end-stage renal disease,^[22,24,25]^ our sample comprised CKM stages 0–3, encompassing those with obesity, metabolic abnormalities, or early renal impairment but no clinical cardiovascular events. Traditionally, these individuals have often been perceived as having relatively low cardiovascular risk. Yet, our findings highlight that significant BAA alone confers a distinctly higher long-term mortality risk. This aligns with growing research focusing on the triple interaction among metabolism-inflammation-aging, suggesting that even at early stages of metabolic derangement, persistent inflammatory stress and immune imbalance may sharply increase long-term susceptibility to cardiovascular and all-cause mortality.^[26]^ Notably, even after adjusting for CKM staging, we continued to observe the independent predictive value of KDM-BAA and PAA. This finding suggests that CKM staging offers a macroscopic view of cardiorenal-metabolic status, whereas BAA provides a microscopic lens on systemic aging processes, such as inflammation and immune dysregulation, so the two measures may complement each other.

Echoing international studies, some researchers have also explored incorporating biological age measures into conventional cardiovascular risk scores to enhance prediction accuracy.^[27,28]^ A similar concept could be extended to CKM staging, which primarily assesses obesity, metabolic risk factors, and kidney function, but may miss high inflammatory burden/high immune activation subpopulations. For these individuals, earlier and more aggressive interventions might yield greater benefits. Overall, this study supports existing evidence on the link between accelerated biological aging and mortality risk and enriches the literature by focusing on the often-overlooked CKM 0–3 stage population. The observed differences in predictive accuracy between PAA and KDM-BAA offer insights for refining future models. Specifically, one could consider integrating biomarkers reflecting inflammation, immunity, and metabolism across multiple pathways, and examining these metrics in broader populations over longer observational periods to determine whether rapid versus slow accelerators show dose-dependent or nonlinear differences in outcomes.

Pathophysiologically, although CKM 0–3 stages lack overt clinical cardiovascular events, multiple cardiometabolic and renal risk factors may already coexist. Chronic low-grade inflammation continuously activates immune cells to release cytokines, injuring the vascular endothelium and nephron structures, and disrupting the balance between cellular apoptosis and regeneration.^[29,30]^ Meanwhile, prolonged metabolic dysfunction, such as insulin resistance or hyperglycemia, exacerbates oxidative stress and thereby accelerates mitochondrial dysfunction and telomere shortening.^[31]^ Excess adipose accumulation and adipose tissue dysfunction also secrete pro-inflammatory mediators, inducing widespread immune imbalances that dysregulate aging pathways such as mechanistic target of rapamycin (mTOR) and AMP-activated protein kinase (AMPK).^[32,33]^ Consequently, at the microscopic level, a vicious cycle of immunosenescence and metabolic disturbance emerges, constituting an inflammation-aging-disease axis manifested macroscopically as accelerated biological aging. Even in individuals without clinical events, this burden of multi-system aging substantially raises the risk of cardiovascular and all-cause mortality.^[34]^ Early identification and mitigation of inflammation and immunosenescence can help disrupt or delay such processes.^[35]^ Moreover, epigenetic modifications, such as DNA methylation and histone modification, may be further amplified by chronic inflammation, thereby aggravating aberrant gene expression and cellular damage.^[36]^ Therefore, in CKM 0–3 stage populations, promptly monitoring and controlling these key pathways might substantially reduce their future burden of cardiovascular and other chronic diseases. This underscores the importance of early intervention.

Our findings offer several implications for clinical practice and public health. First, for early-stage CKM populations (especially stages 0–2, where individuals have obesity or mild metabolic disorders without clinical cardiovascular complications), traditional wisdom typically deems them to have limited cardiovascular risk. Yet if KDM-BAA or PAA reveals marked BAA, these individuals also face a hidden high mortality risk and warrant early lifestyle interventions, tighter metabolic control, and vigilant follow-up. Second, integrating biological age acceleration indices with standard cardio-renal-metabolic measures can inform more comprehensive risk stratification and outcome management. For CKM stages 0 to 3, clinicians should employ multidisciplinary interventions that encompass nutrition, exercise, psychological well-being, and pharmacotherapy to slow aging processes and lessen adverse outcomes.^[37,38]^ Moreover, as precision medicine evolves, measuring biological age has become more accessible. Compared with costly and technically demanding epigenetic assays, KDM-BA and PA computed from conventional biochemical markers are feasible for primary care or large epidemiologic surveys.^[20]^ Such approaches not only enhance clinical decision-making but also hold promise for community-level health promotion. For instance, repeated measurements of biological age (or residuals) before and after interventions may offer a more direct gauge of how a health program impacts the aging process.

This study has several strengths: a large sample size with national representativeness; stringent quality control and standardized methodology in NHANES, allowing reliable measurement of biochemical indices and mortality records; a comprehensive series of subgroup and sensitivity analyses that reinforce the robust predictive value of BAA for mortality. We further built and validated a LASSO-Cox survival model, showing excellent discrimination and calibration, suggesting its potential applicability in both clinical and public health settings. Nevertheless, some limitations exist: First, this is an observational study, and although we adjusted for numerous socioeconomic, lifestyle, and medication confounders, residual confounding or unmeasured factors cannot be fully excluded. Second, we only calculated biological age at baseline and could not track trajectories over time. We also did not differentiate between rapid and stable accelerators with respect to outcomes. Third, some inflammatory or immunological markers, such as interleukin-6 (IL-6) and tumor necrosis factor-alpha (TNF-α), were not consistently measured in NHANES, which prevented us from exploring more detailed inflammatory pathways that might mediate the association between BAA and mortality. Fourth, while CKM staging is widely recognized by the AHA, its applicability in other ethnicities and populations requires further prospective validation. Finally, although weighting, stratification, and multiple sensitivity analyses were applied to minimize selection and information bias, the NHANES design, which combines cross-sectional baseline interviews with prospective mortality linkage, may underestimate variability in individual health status, and self-reported data are prone to recall bias.

Based on our results, several areas warrant further investigation. First, studies with repeated measures over time could clarify whether rapid accelerators differ significantly from slow accelerators in long-term outcomes.^[39]^ Second, combining multi-omics approaches, such as epigenomics, proteomics, and metabolomics, may yield more comprehensive and biologically informative aging clocks.^[40]^ Third, further studies are required to establish the external validity and applicability of BAA metrics across diverse ethnic populations and clinical contexts, such as chronic inflammatory diseases, autoimmune disorders, and malignancies. Finally, rigorously designed randomized controlled trials should investigate whether intensified interventions for people with high BAA can substantially reduce long-term cardiovascular events and mortality, thereby propelling the field of precision medicine forward with stronger evidence.

## 5. Conclusion

In summary, using a large, nationally representative NHANES dataset, we systematically evaluated BAA (KDM-BAA and PAA) among U.S. adults with CKM syndrome stages 0–3 and its association with mortality. Both measures independently predicted all-cause, CVD, and non-CVD mortality, with PAA showing stronger predictive power. These findings remained robust across extensive adjustments, subgroups, and sensitivity analyses. BAA appears partly independent of CKM staging and may signal deeper cardiorenal-metabolic damage and heightened long-term risk. Integrating biological age monitoring with CKM staging could help identify high-risk individuals earlier, enabling targeted interventions and potentially reducing overall mortality. Future large-scale, multi-center studies and intervention trials are needed to validate these results and advance precise prevention and personalized therapy.

## Acknowledgments

We thank Huanxian Liu (Department of Neurology, Chinese PLA General Hospital, Beijing, China), and Haoxian Tang (Department of Clinical Medicine, Shantou University Medical College, Shantou, Guangdong, China) for their valuable comments on the study design and manuscript.

## Author contributions

**Conceptualization:** Xiaoqiang Chen, Yisen Huang, Yingxuan Huang.

**Formal analysis:** Xiaoqiang Chen, Yisen Huang.

**Methodology:** Xiaoqiang Chen.

**Visualization:** Xiaoqiang Chen.

**Writing – original draft:** Xiaoqiang Chen.

**Writing – review & editing:** Xiaoqiang Chen, Yubin Wang, Xiaoqiang Liu.

**Validation:** Yisen Huang, Yingyi Li, Xinqi Chen.

**Software:** Yingxuan Huang.

**Data curation:** Yingyi Li, Xinqi Chen.

**Project administration:** Yubin Wang.

**Supervision:** Xiaoqiang Liu.



















## References

[1] Yang C Li Z Sui Y. Sex differences in cardiovascular-kidney-metabolic risk factors associated with degenerative valvular heart disease. Eur J Prev Cardiol. 2025;32:1705–15. 39937669 10.1093/eurjpc/zwaf050

[2] Ndumele CE Neeland IJ Tuttle KR.; American Heart Association. A synopsis of the evidence for the science and clinical management of Cardiovascular-Kidney-Metabolic (CKM) syndrome: a scientific statement from the American Heart Association. Circulation. 2023;148:1636–64. 37807920 10.1161/CIR.0000000000001186

[3] Lai W Xie Y Zhao X. Elevated systemic immune inflammation level increases the risk of total and cause-specific mortality among patients with chronic kidney disease: a large multi-center longitudinal study. Inflamm Res. 2023;72:149–58. 36352033 10.1007/s00011-022-01659-y

[4] Huang Y Zhou A Huang Y Wang Y Liu X Liu X. Association between phenotypic age and mortality risk in individuals with obesity: a retrospective cohort study. Front Public Health. 2024;12:1505066. 39717032 10.3389/fpubh.2024.1505066 PMC11663737

[5] Hayiroğlu MI Bozbeyoglu E Yildirimtürk O Tekkeşin AI Pehlivanoğlu S. Effect of acute kidney injury on long-term mortality in patients with ST-segment elevation myocardial infarction complicated by cardiogenic shock who underwent primary percutaneous coronary intervention in a high-volume tertiary center. Turk Kardiyol Dern Ars. 2020;48:1–9. 10.5543/tkda.2019.84401 31974325

[6] Hayiroğlu MI Çinar T Çinier G. Cardiac variables associated with atrial fibrillation occurrence and mortality in octogenarians implanted with dual chamber permanent pacemakers. Aging Clin Exp Res. 2022;34:2533–9. 35834163 10.1007/s40520-022-02194-w

[7] Hayiroğlu MI Çinar T Çinier G. Comparison of mortality prediction scores in elderly patients with ICD for heart failure with reduced ejection fraction. Aging Clin Exp Res. 2022;34:653–60. 34424489 10.1007/s40520-021-01960-6

[8] Tang F Yang S Qiu H. Joint association of diabetes mellitus and inflammation status with biological ageing acceleration and premature mortality. Diabetes Metab Syndr. 2024;18:103050. 38833822 10.1016/j.dsx.2024.103050

[9] Wang X Sarker SK Cheng L. Association of dietary inflammatory potential, dietary oxidative balance score and biological aging. Clin Nutr. 2024;43:1–10. 10.1016/j.clnu.2023.11.007 37992632

[10] Wu S Zhu J Lyu S. Impact of DNA-methylation age acceleration on long-term mortality among US adults with cardiovascular-kidney-metabolic syndrome. J Am Heart Assoc. 2025;14:e039751. 40118808 10.1161/JAHA.124.039751 PMC12132836

[11] Chen L Zhao Y Liu F. Biological aging mediates the associations between urinary metals and osteoarthritis among U.S. adults. BMC Med. 2022;20:207. 35710548 10.1186/s12916-022-02403-3 PMC9205020

[12] Liu X Wang Y Huang Y. Association of phenotypic age acceleration with all-cause and cause-specific mortality among U.S. cancer survivors: a retrospective cohort study. BMC Cancer. 2025;25:338. 40001013 10.1186/s12885-025-13760-6 PMC11853897

[13] Zheng Q Cao Z Teng J Lu Q Huang P Zhou J. Association between atherogenic index of plasma with all-cause and cardiovascular mortality in individuals with cardiovascular-kidney-metabolic syndrome. Cardiovasc Diabetol. 2025;24:183. 40287685 10.1186/s12933-025-02742-4 PMC12034140

[14] Tan MY Zhang YJ Zhu SX Wu S Zhang P Gao M. The prognostic significance of stress hyperglycemia ratio in evaluating all-cause and cardiovascular mortality risk among individuals across stages 0-3 of cardiovascular-kidney-metabolic syndrome: evidence from two cohort studies. Cardiovasc Diabetol. 2025;24:137. 40128747 10.1186/s12933-025-02689-6 PMC11934678

[15] Tang H Zhang X Luo N Huang J Zhu Y. Association of dietary live microbes and nondietary prebiotic/probiotic intake with cognitive function in older adults: evidence from NHANES. J Gerontol A Biol Sci Med Sci. 2024;79:glad175. 37480582 10.1093/gerona/glad175

[16] Rattan P Penrice DD Ahn JC. Inverse association of telomere length with liver disease and mortality in the US population. Hepatol Commun. 2022;6:399–410. 34558851 10.1002/hep4.1803 PMC8793996

[17] Liu X Liu X Huang Y. Association between dietary index for gut microbiota and diarrhea among US adults: a cross-sectional analysis of NHANES 2005-2010. Front Nutr. 2025;12:1566314. 40290658 10.3389/fnut.2025.1566314 PMC12021626

[18] Zhang Z Wang P Cui G Li H. Higher HEI-2015 score is associated with reduced risk of fecal incontinence: insights from a large cross-sectional study. BMC Public Health. 2024;24:3221. 39567930 10.1186/s12889-024-20729-w PMC11577588

[19] Wang M Yan H Zhang Y. Accelerated biological aging increases the risk of short- and long-term stroke prognosis in patients with ischemic stroke or TIA. EBioMedicine. 2025;111:105494. 39662178 10.1016/j.ebiom.2024.105494 PMC11697706

[20] Wang H Liu Z Fan H. Association between biological aging and the risk of mortality in individuals with non-alcoholic fatty liver disease: a prospective cohort study. Arch Gerontol Geriatr. 2024;124:105477. 38735225 10.1016/j.archger.2024.105477

[21] Seğmen F Aydemir S Küçük O Dokuyucu R. The roles of vitamin D levels, Gla-Rich Protein (GRP) and Matrix Gla Protein (MGP), and inflammatory markers in predicting mortality in intensive care patients: a new biomarker link? Metabolites. 2024;14:620. 39590856 10.3390/metabo14110620 PMC11596285

[22] Mao R Wang F Zhong Y Meng X Zhang T Li J. Association of biological age acceleration with cardiac morphology, function, and incident heart failure: insights from UK Biobank participants. Eur Heart J Cardiovasc Imaging. 2024;25:1315–23. 38747402 10.1093/ehjci/jeae126

[23] Levine ME Lu AT Quach A. An epigenetic biomarker of aging for lifespan and healthspan. Aging (Albany NY). 2018;10:573–91. 29676998 10.18632/aging.101414 PMC5940111

[24] Zheng G Chang Q Zhang Y. Associations of clinical parameter-based accelerated aging, genetic predisposition with risk of chronic kidney disease and associated life expectancy: a prospective cohort study. Aging Cell. 2025;24:e14453. 39717880 10.1111/acel.14453 PMC11984662

[25] Chen L Yin X Zhao Y. Biological ageing and the risks of all-cause and cause-specific mortality among people with diabetes: a prospective cohort study. J Epidemiol Community Health. 2022;76:771–8. 35738895 10.1136/jech-2022-219142

[26] Li PH Zhang R Cheng LQ Liu JJ Chen HZ. Metabolic regulation of immune cells in proinflammatory microenvironments and diseases during ageing. Ageing Res Rev. 2020;64:101165. 32898718 10.1016/j.arr.2020.101165

[27] Si J Ma Y Yu C.; China Kadoorie Biobank Collaborative Group. DNA methylation age mediates effect of metabolic profile on cardiovascular and general aging. Circ Res. 2024;135:954–66. 39308399 10.1161/CIRCRESAHA.124.325066

[28] Liu CM Kuo MJ Kuo CY. Reclassification of the conventional risk assessment for aging-related diseases by electrocardiogram-enabled biological age. NPJ Aging. 2025;11:7. 39915530 10.1038/s41514-025-00198-0 PMC11802786

[29] Jourde-Chiche N Fakhouri F Dou L. Endothelium structure and function in kidney health and disease. Nat Rev Nephrol. 2019;15:87–108. 30607032 10.1038/s41581-018-0098-z

[30] Oates JC Russell DL Van Beusecum JP. Endothelial cells: potential novel regulators of renal inflammation. Am J Physiol Renal Physiol. 2022;322:F309–21. 35129369 10.1152/ajprenal.00371.2021 PMC8897017

[31] Chaithanya V Kumar J Leela KV Murugesan R Angelin M Satheesan A. Impact of telomere attrition on diabetes mellitus and its complications. Diabetes Epidemiol Manag. 2023;12:100174.

[32] Zhang YX Ou MY Yang ZH Sun Y Li QF Zhou SB. Adipose tissue aging is regulated by an altered immune system. Front Immunol. 2023;14:1125395. 36875140 10.3389/fimmu.2023.1125395 PMC9981968

[33] Xu S Lu F Gao J Yuan Y. Inflammation-mediated metabolic regulation in adipose tissue. Obes Rev. 2024;25:e13724. 38408757 10.1111/obr.13724

[34] Li N Li Y Cui L. Association between different stages of cardiovascular-kidney-metabolic syndrome and the risk of all-cause mortality. Atherosclerosis. 2024;397:118585. 39255681 10.1016/j.atherosclerosis.2024.118585

[35] Perrone MA Aimo A Bernardini S Clerico A. Inflammageing and cardiovascular system: focus on cardiokines and cardiac-specific biomarkers. Int J Mol Sci. 2023;24:844. 36614282 10.3390/ijms24010844 PMC9820990

[36] Switzer CH. Non-canonical nitric oxide signalling and DNA methylation: inflammation induced epigenetic alterations and potential drug targets. Br J Pharmacol. 2026;183:24–39. 38116806 10.1111/bph.16302

[37] Zhu J Yang Y Zeng Y. The association of physical activity behaviors and patterns with aging acceleration: evidence from the UK Biobank. J Gerontol A Biol Sci Med Sci. 2023;78:753–61. 36815559 10.1093/gerona/glad064 PMC10172984

[38] McGee KC Sullivan J Hazeldine J. A combination nutritional supplement reduces DNA methylation age only in older adults with a raised epigenetic age. Geroscience. 2024;46:4333–47. 38528176 10.1007/s11357-024-01138-8 PMC11336001

[39] Li X Ploner A Wang Y. Longitudinal trajectories, correlations and mortality associations of nine biological ages across 20-years follow-up. Elife. 2020;9:e51507. 32041686 10.7554/eLife.51507 PMC7012595

[40] Wu L Xie X Liang T. Integrated multi-omics for novel aging biomarkers and antiaging targets. Biomolecules. 2021;12:39. 35053186 10.3390/biom12010039 PMC8773837

