# Supplementary material for: Biological age acceleration and mortality risk among U.S. adults with cardio-kidney-metabolic syndrome, stages 0–3: An NHANES cohort study
Source: Medicine (Baltimore). 2026 Aug 28;105(35):e50450. doi: 10.1097/MD.0000000000050450 (PMC13529157; doi:10.1097/MD.0000000000050450)
Supplement: Supplementary file 2 [file medi-105-e50450-s002.docx]

**Supplementary Methods Algorithms for KDM–biological and phenotypic age**

1. **The calculation formula for KDM–biological age (BA)**

The values $j$ and $i$ represent the number of biomarkers and the number of samples, respectively. The values $k$, $q$, and $s$ represent the regression slopes, intercepts, and root–mean–square errors of a biomarker regressed on chronological age, respectively. The value $r_{j}^{2}$ denotes the variance explained by regression of chronological age (CA) on biomarkers.

$$BA_{E}=\frac{\Sigma_{\dot{J}=1}^{m}\left( x_{\dot{J}}-q_{j} \right)\left( \frac{k_{j}}{s_{j}^{2}} \right)}{\Sigma_{j=1}^{m}\left( \frac{k_{j}}{s_{j}} \right)^{2}}$$

$$r_{char}=\frac{\Sigma_{j=1}^{m}\frac{r_{j}^{2}}{\sqrt{1-r_{j}^{2}}}}{\Sigma_{j=1}^{m}\frac{r_{j}}{\sqrt{1-{r_{j}}^{2}}}}$$

$$S_{BA}^{2}=\frac{\Sigma_{j=1}^{n}\left( \left( BA_{E_{j}}-CA_{j} \right)-\frac{\Sigma_{ⅈ=1}^{n}(BA_{E_{j}}-CA_{i})}{n} \right)^{2}}{n}-\left( \frac{1-r_{char}^{2}}{r_{char}^{2}} \right)\times(\frac{{({CA}_{max}-{CA}_{min})}^{2}}{12m})$$

$$BA=\frac{\Sigma_{j=1}^{m}\left( x_{j}-q_{j} \right)\left( \frac{k_{j}}{s_{j}^{2}} \right)+\frac{CA}{S_{BA}^{2}}}{\sum_{j=1}^{m} \left( \frac{k_{j}}{s_{j}} \right)^{2}+\frac{1}{S_{BA}^{2}}}$$

2. **The calculation formula for phenotypic age**

$$Phenotypic age = 141.50 + \frac{Ln[-0.00553 \times Ln(exp(\frac{-1.51714 \times\mathrm{ex}p \left( xb \right)}{0.0076927}))]}{0.090165}$$

where *xb* = − 19.907 − 0.0336 × Albumin (g/L) + 0.0095 × Creatinine (μmol/L) + 0.1953 × Glucose (mmol/L) + 0.0954 × LnCRP (mg/dL) − 0.0120 × Lymphocyte Percent (%) + 0.0268 × Mean Cell Volume (fL) + 0.3306 × Red Cell Distribution Width (%) + 0.00188 × Alkaline Phosphatase (U/L) + 0.0554 × White Blood Cell Count (1000 cells/ μL) + 0.0804 × chronological age (years).
