# Supplementary material for: Biological age acceleration and mortality risk among U.S. adults with cardio-kidney-metabolic syndrome, stages 0–3: An NHANES cohort study
Source: Medicine (Baltimore). 2026 Aug 28;105(35):e50450. doi: 10.1097/MD.0000000000050450 (PMC13529157; doi:10.1097/MD.0000000000050450)
Supplement: Supplementary file 3 [file medi-105-e50450-s004.docx]

**Supplementary Table 2 Weighted adjusted hazard ratios of biological aging with risk of all–cause and cause–specific mortality after excluding deaths within 2 years**

| **Characteristics** | **Crude model** |  |  | **Model 1** |  |  | **Model 2** |  |  | **Model 3** |  |
| --- | --- | --- | --- | --- | --- | --- | --- | --- | --- | --- | --- |
|  | **HR (95% CI)** | ***P* value** |  | **HR (95% CI)** | ***P* value** |  | **HR (95% CI)** | ***P* value** |  | **HR (95% CI)** | ***P* value** |
| **All–cause mortality** |  |  |  |  |  |  |  |  |  |  |  |
| KDM–BAA residual | 1.01 (0.99, 1.03) | 0.399 |  | 1.05 (1.04, 1.06) | <0.001 |  | 1.05 (1.04, 1.06) | <0.001 |  | 1.04 (1.03, 1.05) | <0.001 |
| KDM–BAA |  |  |  |  |  |  |  |  |  |  |  |
| No | Reference |  |  | Reference |  |  | Reference |  |  | Reference |  |
| Yes | 0.96 (0.87, 1.06) | 0.443 |  | 1.48 (1.35, 1.62) | <0.001 |  | 1.43 (1.30, 1.57) | <0.001 |  | 1.35 (1.23, 1.49) | <0.001 |
| PAA residual | 1.04 (1.04, 1.05) | <0.001 |  | 1.04 (1.04, 1.05) | <0.001 |  | 1.04 (1.03, 1.04) | <0.001 |  | 1.04 (1.03, 1.04) | <0.001 |
| PAA |  |  |  |  |  |  |  |  |  |  |  |
| No | Reference |  |  | Reference |  |  | Reference |  |  | Reference |  |
| Yes | 2.76 (2.47, 3.09) | <0.001 |  | 2.34 (2.10, 2.61) | <0.001 |  | 2.04 (1.83, 2.28) | <0.001 |  | 1.95 (1.75, 2.17) | <0.001 |
| **CVD mortality** |  |  |  |  |  |  |  |  |  |  |  |
| KDM–BAA residual | 1.04 (1.01, 1.07) | 0.011 |  | 1.08 (1.06, 1.10) | <0.001 |  | 1.07 (1.05, 1.09) | <0.001 |  | 1.06 (1.03, 1.08) | <0.001 |
| KDM–BAA |  |  |  |  |  |  |  |  |  |  |  |
| No | Reference |  |  | Reference |  |  | Reference |  |  | Reference |  |
| Yes | 1.25 1.04, 1.50) | 0.018 |  | 2.08 (1.73, 2.49) | <0.001 |  | 1.91 (1.57, 2.33) | <0.001 |  | 1.74 (1.44, 2.10) | <0.001 |
| PAA residual | 1.04 (1.04, 1.05) | <0.001 |  | 1.04 (1.04, 1.05) | <0.001 |  | 1.04 (1.03, 1.04) | <0.001 |  | 1.03 (1.02, 1.04) | <0.001 |
| PAA |  |  |  |  |  |  |  |  |  |  |  |
| No | Reference |  |  | Reference |  |  | Reference |  |  | Reference |  |
| Yes | 2.95 (2.47, 3.53) | <0.001 |  | 2.46 (2.05, 2.94) | <0.001 |  | 2.03 (1.68, 2.46) | <0.001 |  | 1.78 (1.46, 2.16) | <0.001 |
| **Non–CVD mortality** |  |  |  |  |  |  |  |  |  |  |  |
| KDM–BAA residual | 1.00 (0.97, 1.02) | 0.725 |  | 1.04 (1.03, 1.05) | <0.001 |  | 1.04 (1.03, 1.05) | <0.001 |  | 1.04 (1.02, 1.05) | <0.001 |
| KDM–BAA |  |  |  |  |  |  |  |  |  |  |  |
| No | Reference |  |  | Reference |  |  | Reference |  |  | Reference |  |
| Yes | 0.87 (0.77, 0.98) | 0.020 |  | 1.30 (1.18, 1.44) | <0.001 |  | 1.28 (1.16, 1.42) | <0.001 |  | 1.24 (1.12, 1.37) | <0.001 |
| PAA residual | 1.04 (1.04, 1.05) | <0.001 |  | 1.04 (1.04, 1.05) | <0.001 |  | 1.04 (1.03, 1.04) | <0.001 |  | 1.04 (1.03, 1.04) | <0.001 |
| PAA |  |  |  |  |  |  |  |  |  |  |  |
| No | Reference |  |  | Reference |  |  | Reference |  |  | Reference |  |
| Yes | 2.69 (2.36, 3.07) | <0.001 |  | 2.30 (2.02, 2.62) | <0.001 |  | 2.05 (1.80, 2.34) | <0.001 |  | 2.02 (1.77, 2.30) | <0.001 |

Crude model: no other covariates were adjusted

Model 1: Adjusted for age and sex.

Model 2: Further adjusted for race, marital status, PIR, education, BMI, smoking status, alcohol intake, physical activity, and HEI–2015.

Model 3: Further adjusted for antidiabetic drugs, antihyperlipidemic drugs, and antihypertensive drugs.

Abbreviations: PIR, poverty–income ratio; BMI, body mass index; HEI–2015, Healthy Eating Index–2015; KDM–BAA, Klemera–Doubal Method Biological Age Acceleration; PAA, phenotypic age acceleration; CKM, cardiovascular–kidney–metabolic.
