# Supplementary material for: Biological age acceleration and mortality risk among U.S. adults with cardio-kidney-metabolic syndrome, stages 0–3: An NHANES cohort study
Source: Medicine (Baltimore). 2026 Aug 28;105(35):e50450. doi: 10.1097/MD.0000000000050450 (PMC13529157; doi:10.1097/MD.0000000000050450)
Supplement: Supplementary file 4 [file medi-105-e50450-s005.docx]

**Supplementary Table 3 Weighted adjusted hazard ratios of biological aging with risk of all–cause and cause–specific mortality after further adjusting for CKM stage**

| **Characteristics** | **Crude model** |  |  | **Model 1** |  |  | **Model 2** |  |  | **Model 3** |  |
| --- | --- | --- | --- | --- | --- | --- | --- | --- | --- | --- | --- |
|  | **HR (95% CI)** | ***P* value** |  | **HR (95% CI)** | ***P* value** |  | **HR (95% CI)** | ***P* value** |  | **HR (95% CI)** | ***P* value** |
| **All–cause mortality** |  |  |  |  |  |  |  |  |  |  |  |
| KDM–BAA residual | 1.01 (1.00, 1.03) | 0.097 |  | 1.05 (1.04, 1.06) | <0.001 |  | 1.05 (1.04, 1.06) | <0.001 |  | 1.04 (1.03, 1.05) | <0.001 |
| KDM–BAA |  |  |  |  |  |  |  |  |  |  |  |
| No | Reference |  |  | Reference |  |  | Reference |  |  | Reference |  |
| Yes | 1.00 (0.91, 1.10) | 0.956 |  | 1.53 (1.41, 1.66) | <0.001 |  | 1.48 (1.35, 1.62) | <0.001 |  | 1.34 (1.22, 1.46) | <0.001 |
| PAA residual | 1.04 (1.04, 1.05) | <0.001 |  | 1.04 (1.04, 1.05) | <0.001 |  | 1.04 (1.03, 1.04) | <0.001 |  | 1.03 (1.03, 1.04) | <0.001 |
| PAA |  |  |  |  |  |  |  |  |  |  |  |
| No | Reference |  |  | Reference |  |  | Reference |  |  | Reference |  |
| Yes | 2.84 (2.55, 3.17) | <0.001 |  | 2.40 (2.16, 2.66) | <0.001 |  | 2.11 (1.89, 2.35) | <0.001 |  | 1.97 (1.77, 2.20) | <0.001 |
| **CVD mortality** |  |  |  |  |  |  |  |  |  |  |  |
| KDM–BAA residual | 1.04 (1.02, 1.07) | 0.001 |  | 1.08 (1.06, 1.10) | <0.001 |  | 1.07 (1.05, 1.09) | <0.001 |  | 1.06(1.04, 1.08) | <0.001 |
| KDM–BAA |  |  |  |  |  |  |  |  |  |  |  |
| No | Reference |  |  | Reference |  |  | Reference |  |  | Reference |  |
| Yes | 1.24 (1.04, 1.48) | 0.016 |  | 2.04 (1.72, 2.41) | <0.001 |  | 1.89 (1.58, 2.28) | <0.001 |  | 1.68 (1.41, 2.00) | <0.001 |
| PAA residual | 1.04 (1.04, 1.05) | <0.001 |  | 1.04 (1.04, 1.05) | <0.001 |  | 1.04 (1.03, 1.04) | <0.001 |  | 1.03 (1.02, 1.04) | <0.001 |
| PAA |  |  |  |  |  |  |  |  |  |  |  |
| No | Reference |  |  | Reference |  |  | Reference |  |  | Reference |  |
| Yes | 2.92 (2.44, 3.48) | <0.001 |  | 2.40 (2.00, 2.88) | <0.001 |  | 2.00 (1.65, 2.43) | <0.001 |  | 1.74 (1.43, 2.12) | <0.001 |
| **Non–CVD mortality** |  |  |  |  |  |  |  |  |  |  |  |
| KDM–BAA residual | 1.00 (0.98, 1.02) | 0.815 |  | 1.04 (1.03, 1.06) | <0.001 |  | 1.04 (1.03, 1.06) | <0.001 |  | 1.03 (1.02, 1.05) | <0.001 |
| KDM–BAA |  |  |  |  |  |  |  |  |  |  |  |
| No | Reference |  |  | Reference |  |  | Reference |  |  | Reference |  |
| Yes | 0.92 (0.83, 1.02) | 0.129 |  | 1.38 (1.25, 1.51) | <0.001 |  | 1.36 (1.23, 1.49) | <0.001 |  | 1.23 (1.12, 1.36) | <0.001 |
| PAA residual | 1.04 (1.04, 1.05) | <0.001 |  | 1.04 (1.04, 1.05) | <0.001 |  | 1.04 (1.03, 1.04) | <0.001 |  | 1.04 (1.03, 1.04) | <0.001 |
| PAA |  |  |  |  |  |  |  |  |  |  |  |
| No | Reference |  |  | Reference |  |  | Reference |  |  | Reference |  |
| Yes | 2.81 (2.48, 3.18) | <0.001 |  | 2.39 (2.12, 2.70) | <0.001 |  | 2.15 (1.90, 2.43) | <0.001 |  | 2.07 (1.82, 2.34) | <0.001 |

Crude model: no other covariates were adjusted

Model 1: Adjusted for age and sex.

Model 2: Further adjusted for race, marital status, PIR, education, BMI, smoking status, alcohol intake, physical activity, and HEI–2015.

Model 3: Further adjusted for antidiabetic drugs, antihyperlipidemic drugs, and antihypertensive drugs.

Abbreviations: PIR, poverty–income ratio; BMI, body mass index; HEI–2015, Healthy Eating Index–2015; KDM–BAA, Klemera–Doubal Method Biological Age Acceleration; PAA, phenotypic age acceleration; CKM, cardiovascular–kidney–metabolic.
